# Automated Detection of Corneal Ulcer Using Combination Image Processing and Deep Learning

**DOI:** 10.3390/diagnostics12123204

**Published:** 2022-12-17

**Authors:** Isam Abu Qasmieh, Hiam Alquran, Ala’a Zyout, Yazan Al-Issa, Wan Azani Mustafa, Mohammed Alsalatie

**Affiliations:** 1 Biomedical Systems and Medical Informatics Engineering, Yarmouk University, Irbid 21163, Jordan; 2 Department of Computer Engineering, Yarmouk University, Irbid 21163, Jordan; 3 Faculty of Electrical Engineering & Technology, Campus Pauh Putra, Universiti Malaysia Perlis (UniMAP), Arau, Perlis 02600, Malaysia; 4 Advanced Computing (AdvComp), Centre of Excellence (CoE), Campus Pauh Putra, Universiti Malaysia Perlis (UniMAP), Arau, Perlis 02600, Malaysia; 5 The Institute of Biomedical Technology, King Hussein Medical Center, Royal Jordanian Medical Service, Amman 11855, Jordan

**Keywords:** cornel ulcer, Hough transform, semantic segmentation, ResNet18, localization

## Abstract

A corneal ulcers are one of the most common eye diseases. They come from various infections, such as bacteria, viruses, or parasites. They may lead to ocular morbidity and visual disability. Therefore, early detection can reduce the probability of reaching the visually impaired. One of the most common techniques exploited for corneal ulcer screening is slit-lamp images. This paper proposes two highly accurate automated systems to localize the corneal ulcer region. The designed approaches are image processing techniques with Hough transform and deep learning approaches. The two methods are validated and tested on the publicly available SUSTech-SYSU database. The accuracy is evaluated and compared between both systems. Both systems achieve an accuracy of more than 90%. However, the deep learning approach is more accurate than the traditional image processing techniques. It reaches 98.9% accuracy and Dice similarity 99.3%. However, the first method does not require parameters to optimize an explicit training model. The two approaches can perform well in the medical field. Moreover, the first model has more leverage than the deep learning model because the last one needs a large training dataset to build reliable software in clinics. Both proposed methods help physicians in corneal ulcer level assessment and improve treatment efficiency.

## 1. Introduction

A corneal ulcer is a type of illness in the cornea; it comes from infection or injury and leads to ocular morbidity [1,2]. The likelihood of vision impairment is decreased by early identification and differentiation of various ulcer conditions. Slit-lamp imaging techniques used in conventional clinical procedures can be tedious, costly, and time-consuming. The following issues make it challenging to appropriately segment corneal ulcers: significant discrepancies in the pathological morphologies of point-flaky and flaky corneal ulcers, hazy border, noise interference, and a dearth of reliable ground-truth slit-lamp pictures. To recognize and quantify corneal ulcers from ocular staining pictures, various segmentation procedures are needed. Due to the varied sizes and forms of point-flaky mixed corneal ulcers and flaky corneal ulcers, it is difficult to segment them in a slit-lamp picture. The lack of high-quality datasets for both corneal ulcers and their ground truth segment, particularly for supervised learning-based segmentation algorithms, has hampered the development of such systems [3,4]. Corneal segmentation is the first step for diagnosing and assessing ocular surface damage. Therefore, extracting information from fluorescein images is a big challenge for specialists. However, the automated method may help the specialist in localizing and extracting the corneal ulcer region for further assessment. This paper proposed two methods for corneal ulcer segmentation: image processing techniques and semantic segmentation using deep learning. Section 2 is devoted to the most recent studies on ulcer segmentation approaches.

## 2. Review of the Study

In 2018, Lijie Deng et al. proposed a pipeline for automatically extracting corneal ulcers that uses machine learning and image processing techniques based on fluorescein staining images. Each image was segmented using simple linear iterative clustering, and a support vector machine discriminated between the two classes, followed by erosion and dilation procedures to polish the images. The suggested method achieved a mean accuracy of 98.4%, significantly outperforming Otsu thresholding and active contour techniques. The problem with this study is that the suggested model is semiautomatic since it uses manually labeled landmarks [5]. In 2019, Zhenrong Liu et al. developed an automatic pipeline for segmenting flaky corneal ulcers from fluorescein staining images. They employed a combination of Gaussian Mixture Models (GMM) and Otsu thresholding. They employed the HSV color space, and the number of Gaussian was determined using information theory. The model was validated using 150 images and achieved a Dice similarity coefficient of 0.88 [6].

In 2020, Jessica Loo et al. developed SLIT-Net, an automatic algorithm for the segmentation of microbial keratitis biomarkers under two different illuminations. SLIT-Net segments and identifies four pathological ROIs on diffuse white light images, one pathological ROI on diffuse blue light images, and two pathological ROIs on all images. The model was tested using manually annotated slit lamp photographs from 133 eyes. They used seven-fold cross-validation and achieved a Dice that ranged between 0.62–0.95 for all ROIs [7]. Additionally, in 2020, Pablo Lima et al. suggested a semiautomatic approach using supervised machine learning and image processing techniques to segment corneal lesions. They evaluated the multi-layer perceptron, SVM, K-nearest neighbors, and random forest algorithms. Random forest outperformed all other algorithms, achieving a Dice similarity of 0.85 and an accuracy of 99.08% [8]. Finally, Junyan Lyn et al. proposed a novel transfer learning-based model for corneal segmentation using 712 images from the publicly available SUSTech-SYSU dataset. The suggested model contained an encoder-decoder with an Xception feature extractor using atrous spatial pyramid pooling. The proposed method achieved a Dice score of 0.9582, 97.63% accuracy, and 95.37% sensitivity [9].

In 2021, Veena Mayya et al. [10] developed a multi-scale convolutional neural network (MS-CNN) for accurate corneal segmentation. The suggested model consisted of a deep neural pipeline to automatically segment images followed by a ResNeXt for differentiation. The authors successfully detected fungal keratitis with an 88.96% accuracy using 133 images from the Loo et al. dataset [7]. Additionally, in 2021, Tingting Wang et al. proposed a novel Corneal Ulcer Segmentation Network (CU-SegNet) to segment corneal ulcers with different shapes and sizes in fluorescein images. They used a U-shape encoder-decoder structure and two novel modules. To demonstrate their network effectiveness, the proposed network was evaluated on the SUSTech-SYSU dataset and achieved a Dice coefficient of 0.8914 [11]. To improve the segmentation accuracy further, in 2022, the same research group developed a novel semi-supervised multi-scale self-transformer Generative Adversarial Network (Semi-MsST-GAN) for corneal ulcer segmentation in slit lamp images. Again, they evaluated their model using the SUSTech-SYSU dataset and achieved better segmentation performance than the state-of-the-art CNN-based methods. However, the limited number of slit lamp images available for training and evaluation represents a limitation for both studies [12].

This paper compares the effectiveness of employing image processing techniques and deep learning approaches on corneal ulcer region segmentation. Section 3 presents the two proposed methods, while Section 4 illustrates the results and discusses the performance of each method in terms of accuracy, sensitivity, and specificity. On the other hand, Section 5 is devoted to the conclusion and future work.

## 3. Materials and Methods

This paper proposes two methods for the automatic segmentation of corneal ulcers. The first method is image processing techniques, and the second is the semantic segmentation method. The dataset utilized in this paper is the publicly available SUSTech-SYSU database [13,14,15]. The dataset consists of 712 fluorescein-stained images that acquired the ocular surface region for patients with different corneal ulcer disease levels. In addition, there are 354 images labeled where the corneal ulcer region is localized. The labeled images are used for evaluating both methods. On top of that, they are used for building deep learning models in the semantic segmentation procedure. The corresponding sections clarify the proposed methods.

### 3.1. Image Processing with Hough Transform

**Figure 1:**
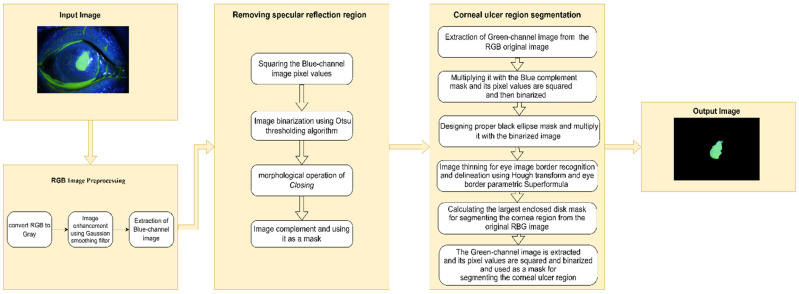
The proposed method block diagram.

The first method utilizes the benefits of image processing techniques with the Hough transform to segment the corneal ulcer region. The designed method is shown in Figure 1.

**Figure 2:**
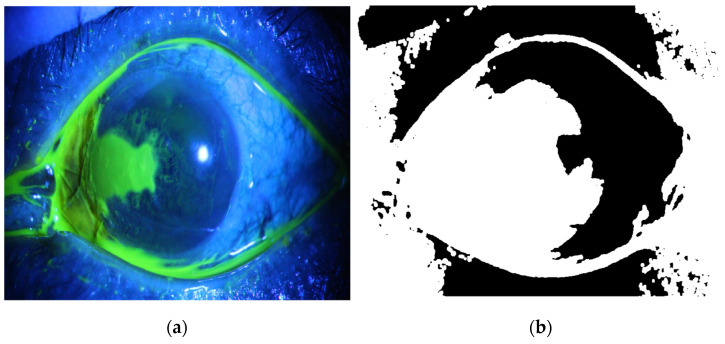
(**a**) The original colored image (**b**). The specular reflection mask.

The corneal ulcer region segmentation system proposed in this work is fully automated. Segmentation of the corneal ulcer regions from the whole RGB eye image undergoes several stages. First, the image is subjected to preprocessing stage by initially excluding most unwanted details from the image, particularly the specular reflection region. This is performed by taking the blue part of the image, then squaring its pixel values and binarizing the output. Next, we applied the morphological operation of closing, followed by calculating its complement, as illustrated in Figure 2, for one of the corneal ulcer image datasets, as an example.

**Figure 3:**
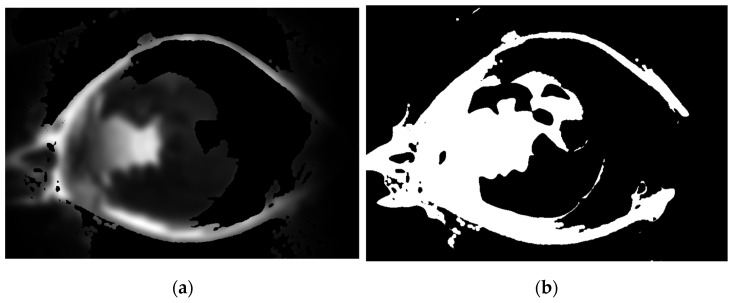
(**a**). The green part smoothed image after masking (**b**). The binarized image of (**a**).

The binary image shown in Figure 2b was then multiplied by the green part of the original-colored image after smoothing using a Gaussian filter, which gives the output shown in Figure 3a. The pixel values are then squared and binarized to give the image shown in Figure 3b.

**Figure 4:**
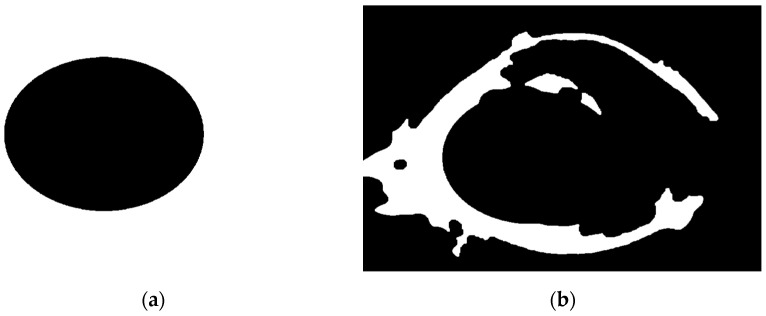
(**a**) The ellipse mask, (**b**) the binarized image of Figure 3, (**b**) after closing and masking with ellipse mask.

**Figure 5:**
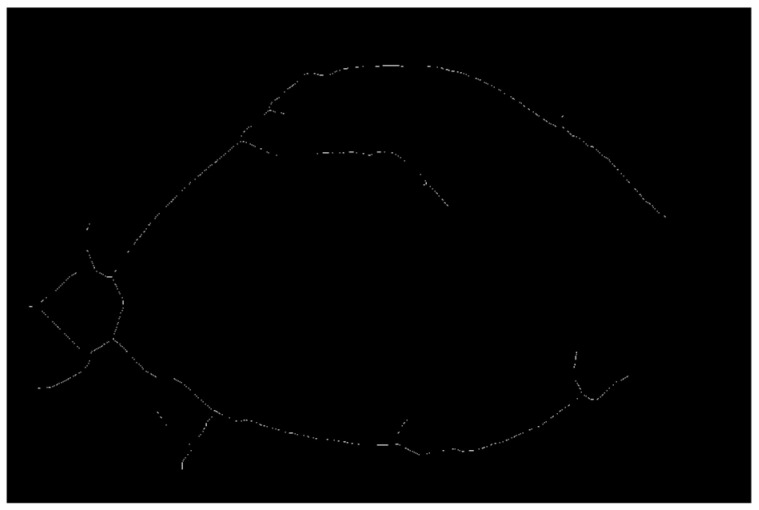
After applying image thinning to the image shown in Figure 4b.

Next, designing an ellipse mask with proper semi-minor and major axis and centroid coordinates is similar to the binary image shown in Figure 3b. The mask shown in Figure 3a is used to exclude most of the unwanted details by multiplying the mask with the binary image shown in Figure 2b, which then gives the image shown in Figure 4b. The final step of the preprocessing stage is performing a thinning operation on the image shown in Figure 4b, which gives the image shown in Figure 5.

**Figure 6:**
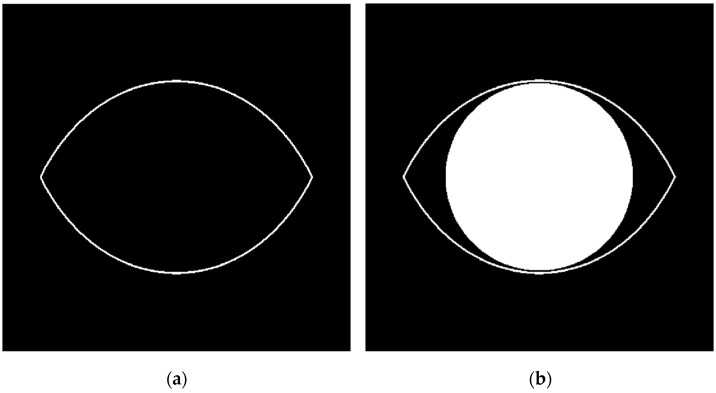
(**a**). Eye border model using Superformula with proper parameters. (**b**). The enclosed disk should separate the cornea region.

**Figure 7:**
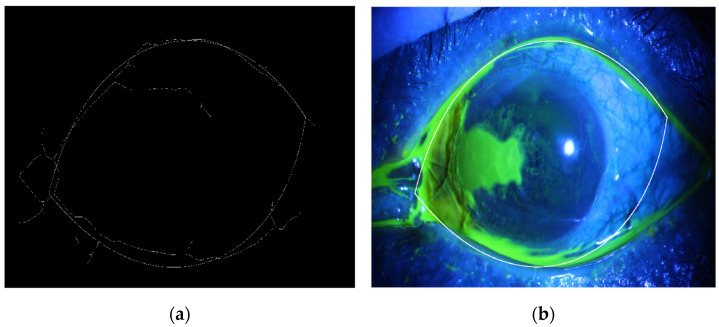
(**a**). The eye border recognition using the Superformula shape model and Hough transform, (**b**). The enclosed recognized eye border with the original image for illustration.

**Figure 8:**
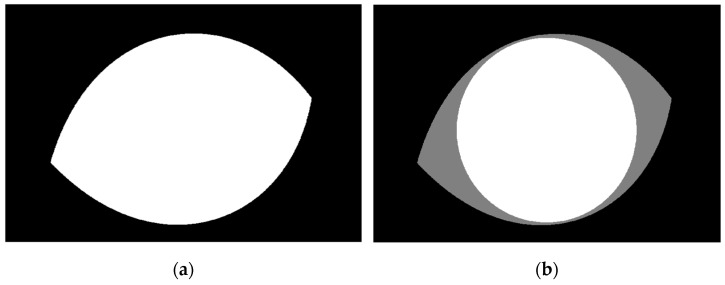
(**a**) The filled recognized eye border in the adopted example. (**b**) The enclosed disk is used as a mask to separate the cornea region.

In general, the eye contour extraction shown in Figure 5 is insufficiently accurate due to the many details in the eye image. To make a better delineation of the eye border, we performed the second stage, which is eye border recognition using a proper eye border mathematical model, and then used a proper recognition algorithm. Hough transform was used as a parametric shape recognition algorithm, where the eye border parametric shape was generated using a closed mathematical formula introduced by Johan Gielis, namely the Superformula [16]. It models curves called Gielis curves, as described by the polar coordinate, r(ϕ), in the corresponding equation

(1) $$r\left(\phi \right)=\frac{1}{{\left[{\left(\left|\frac{1}{a}\cos\left(\phi \frac{m}{4}\right)\right|\right)}^{n_{2}}+{\left(\left|\frac{1}{b}\sin\left(\phi \frac{m}{4}\right)\right|\right)}^{n_{3}}\right]}^{1/n_{1}}}\;,$$

where *r* is the radial distance to the origin, ϕ is the polar angle, and the rational number m is the value of rotational symmetry. The exponents $n_{1}$, $n_{2},$ and $n_{3}$ are introduced, which, with the m parameter, allow a greater degree of freedom and enable the Superformula equation to represent several useful shapes. The chosen parameters for mimicking the eye border are 1, 1, 1, and 2 for $n_{1}$, $n_{2}$, $n_{3}$, and m, respectively, which gives the shape shown in Figure 6a. To determine the iris region, where the cornea is positioned directly in front of the iris and pupil, a disk is designed with a diameter and centroid equal to the semi-minor and centers of the eye-recognized shape respectively, as shown in Figure 5b. By applying this concept to the eye image border and cornea region in the adopted corneal ulcer image sample, we get the output shown in Figure 6 and Figure 7, respectively. Next, the ulcer region of interest is separated by multiplying the mask shown in image Figure 2b with the image shown in Figure 8a to get the image shown in Figure 8b.

**Figure 9:**
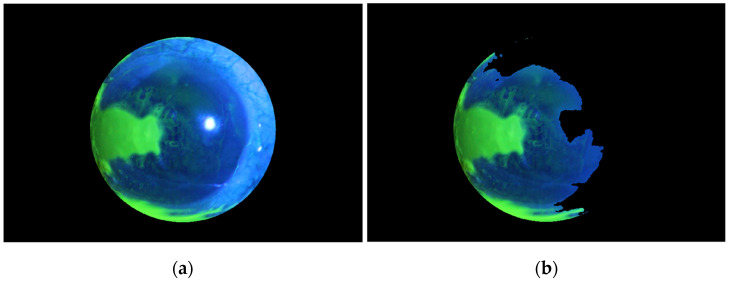
(**a**). Separation of the cornea region using the recognized disk mask. (**b**) The separated cornea region after masking with a specular reflection mask.

**Figure 10:**
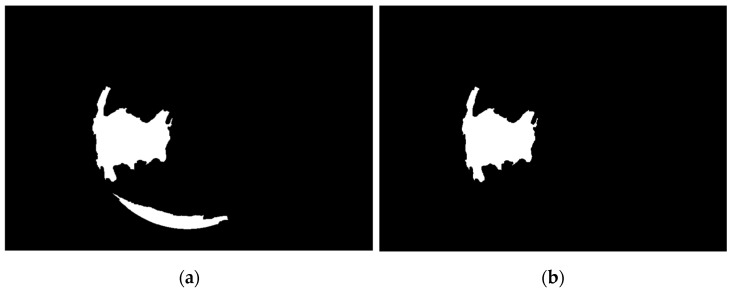
(**a**). Two potential corneal ulcer segments mask (**b**). The mask’s segment connected to the recognized eye border with a ratio of semi-major to semi-minor is greater than the predefined threshold being excluded.

**Figure 11:**
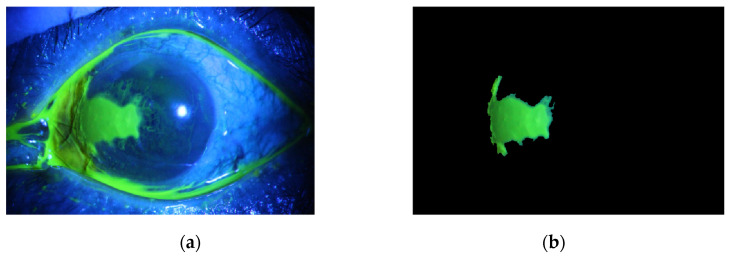
(**a**) The original image. (**b**) After masking the original image with the corneal ulcer mask shown in the image of Figure 10b.

The pixel values of the green part of the image shown in Figure 9b are squared and binarized, yielding the image shown in Figure 10a. The mask segments shown are tested in the segmentation system. Provided the segment is connected to the eye border in which its semi-major to semi-minor ratio is greater than a certain threshold, it will be considered as an accumulation of the fluorescein stain at the eyelids. It will then be excluded from the final ulcer regions result, as shown in Figure 10b. Finally, the original image will be masked with the remaining mask segments, as in the result shown in Figure 11.

### 3.2. Semantic Segmentation

**Figure 12:**
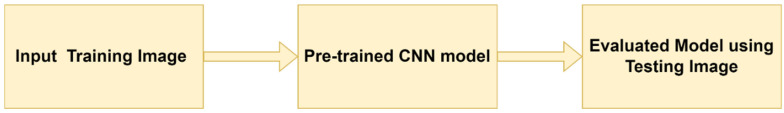
Deep learning method for ulcer localization.

The second method that is proposed in this paper is semantic segmentation. Figure 12 demonstrates the steps for automated segmentation using a deep learning model.

As stated in Figure 12, the system splits the dataset (images and their labels) into training and test partitions. The pre-trained convolutional network in this paper is ResNet 18 [15,17]. The pre-trained CNN model was trained and evaluated on the test data.

Semantic segmentation divides image pixels into one or more semantically interpretable classes rather than real-world objects. Region proposal and annotation is the process of categorizing pixel values into distinct groups using CNN. Candidate object patches (COMPs) are small groups of pixels that most likely belong to the same object as region proposals.

The semantic segmentation procedure is started by the encoder network and followed by the decoder network. The encoder is typically a pre-trained network such as ResNets, which is followed by a decoder network. The type of ResNet used in this paper is the Resnet-18 model that won the 2016 ImageNet competition. It is well-known due to its depth and use of residual blocks [18]. These blocks are essential for solving obstacle issues in training by introducing identity skip connections, which allow layers to copy their inputs to the next layer [19].

**Figure 13:**
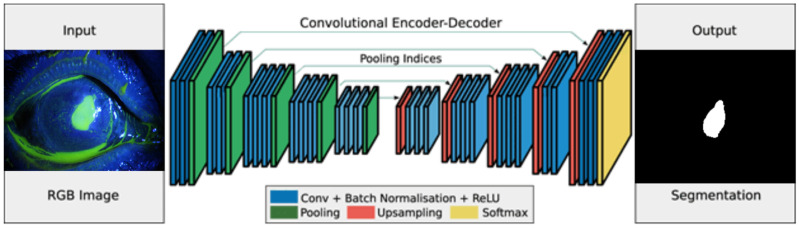
Deep learning method for ulcer localization [20].

To create a segmentation map, encoders may be convolutional neural networks, and decoders may be based on deconvolutional or transposed neural networks [20,21]. Figure 13 describes the procedure of semantic segmentation, which is based mainly on the deep learning approach [22]. The corresponding figure illustrates that the input image passes through a trained deep-learning model to end by the localization of the ulcer region.

The pre-trained ResNet18 was used, and the data were divided into 70% training and 30% testing. The images were resized to 224 × 224 × 3 to match the input requirements for the first layer in ResNet18. The model was trained using MATLAB^®^ with a single CPU. The hyper-parameters are the Adam optimization method besides the initial learning rate of 0.0001, with a minibatch size of 32 and a maximum epoch of 50.

## 4. Results and Discussion

Both methods are applied to the whole dataset, trained, validated, and tested to localize ulcer regions in the cornea.

### 4.1. Image Processing and Hough Transform

**Figure 14:**
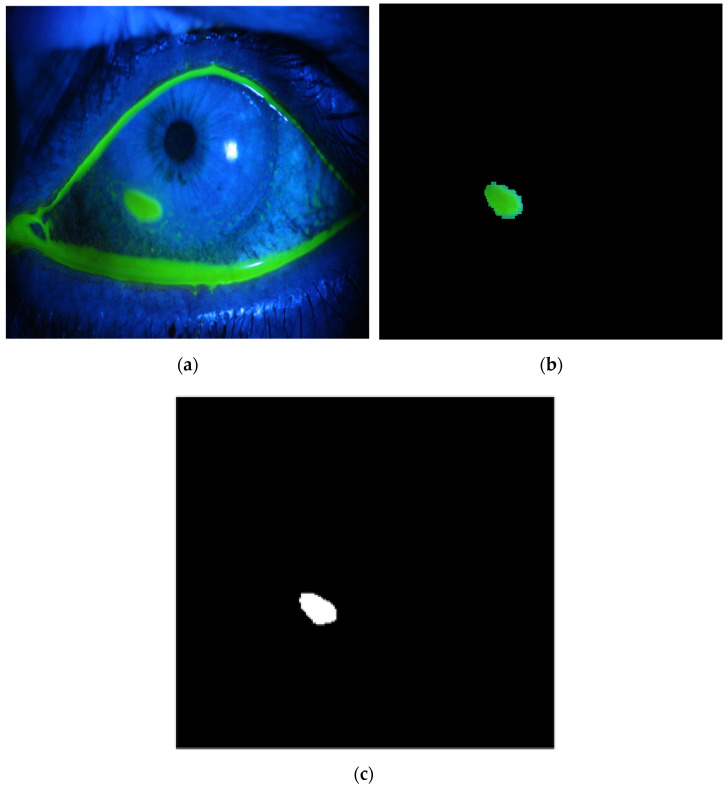
Example 1: Comparison between segmentation output and the ground truth (**a**) original image, (**b**) the segmentation of ulcer region using the first proposed method, (**c**) and the ground truth of the corresponding input image.

**Figure 15:**
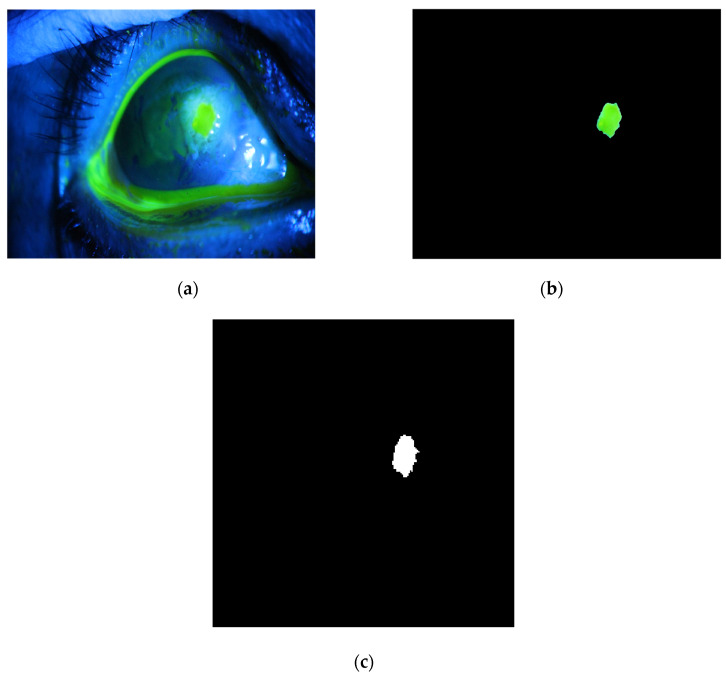
Example 2: Comparison between segmentation output and the ground truth (**a**) original image, (**b**) the segmentation of ulcer region using the first proposed method, (**c**) and the ground truth of the corresponding input image.

**Figure 16:**
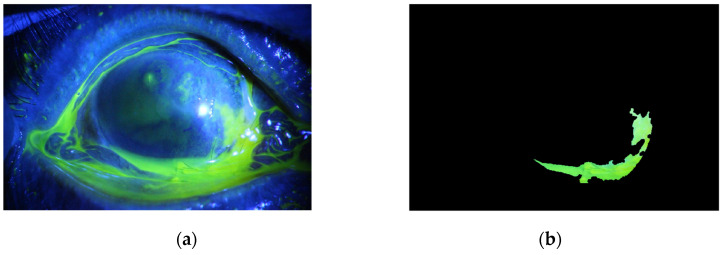

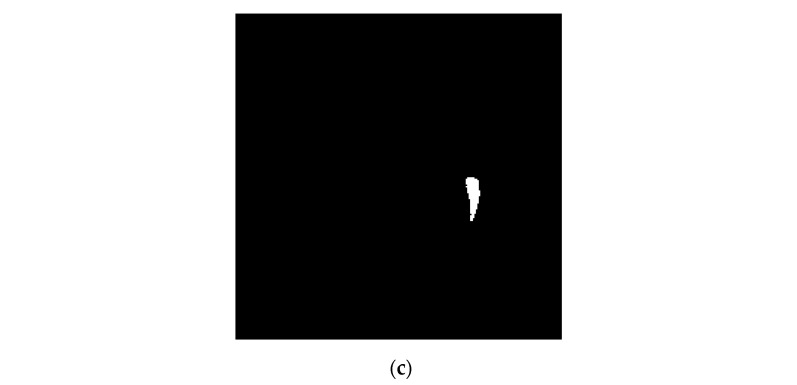
Example 3: Comparison between segmentation output and the ground truth (**a**) original image, (**b**) the segmentation of ulcer region using the first proposed method, (**c**) and the ground truth of the corresponding input image.

**Figure 17:**
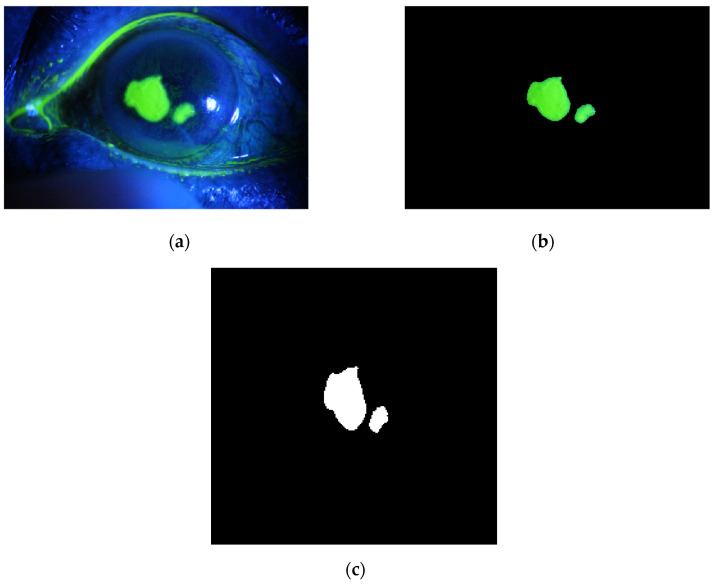
Example 4: Comparison between segmentation output and the ground truth (**a**) original image, (**b**) the segmentation of ulcer region using the first proposed method, (**c**) and the ground truth of the corresponding input image.

**Figure 18:**
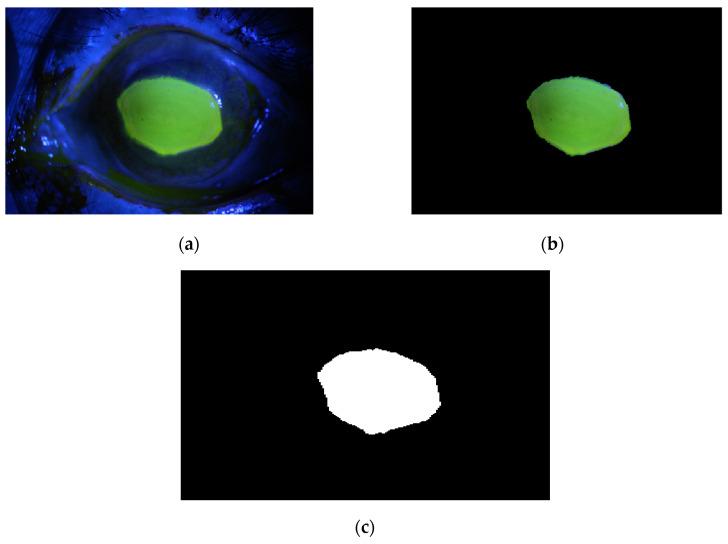
Example 5: Comparison between segmentation output and the ground truth (**a**) original image, (**b**) the segmentation of ulcer region using the first proposed method, (**c**) and the ground truth of the corresponding input image.

The method is applied to whole images. Figure 14, Figure 15, Figure 16, Figure 17 and Figure 18 depict some of the obtained results for different shapes of ulcer regions. Each figure illustrates the original image, the segmentation output, and its corresponding ground truth.

The examples of figures from Figure 14, Figure 15, Figure 16, Figure 17 and Figure 18 illustrate the output of the first proposed method. All figures describe the ability of the proposed method to localize the ulcer region with high similarities to the ground truth. Similarity indices are calculated for each case, such as the Jaccard similarity index and intersection union unit (IOU). The similarities indices are almost 100% for all presented images except the image in Figure 16. As shown in Figure 16, the method was sensitive to the bottom region of the eye to detect ulcer region that is not presented in the ground truth. In this case, the Jaccard and IOU indices are too low. However, the proposed method may have the capability to distinguish ulcer regions from other eye regions more than manual segmentation.

### 4.2. Semantic Segmentation

After training the model on 70% of the whole dataset, accuracy, sensitivity, and specificity were calculated for the training and test stages. The accuracy reveals the percentage of correctly classified pixels to all over pixels. Table 1 describes the results of sensitivity, accuracy, and specificity of semantic deep learning segmentation for both training and test stages [23,24,25,26,27,28,29,30,31].

$$Accuracy=\frac{TP+TN}{TP+TN+FP+FN}$$

$$Specificity=\frac{TN}{FP+TN}$$

$$Sensitivity=\frac{TP}{TP+FN}$$

**Figure 19:**
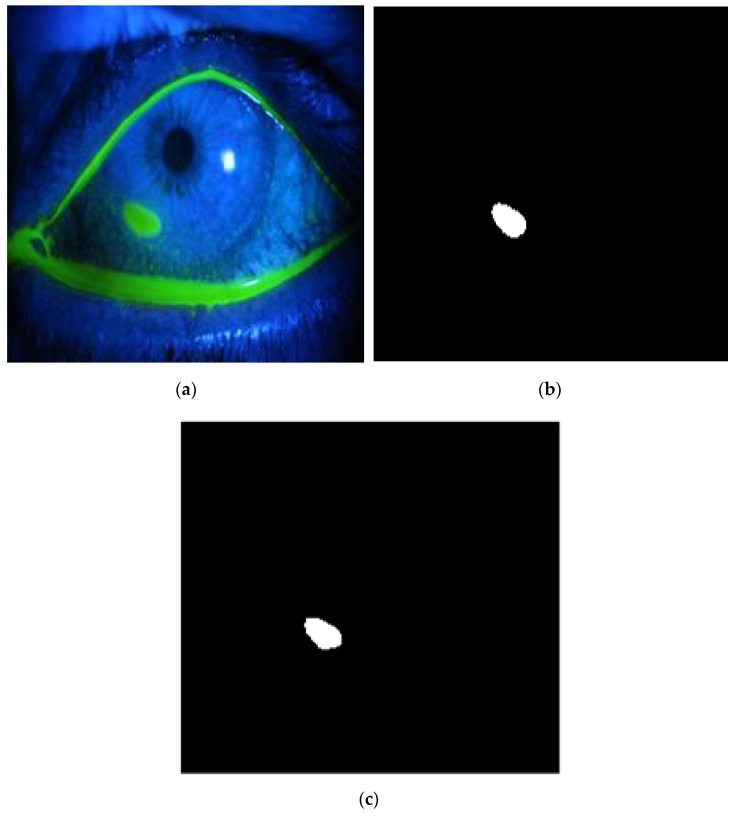
Example 1: Semantic segmentation approach, (**a**) the original image, (**b**) the segmentation output, (**c**) and the ground truth of the corresponding input image.

**Figure 20:**
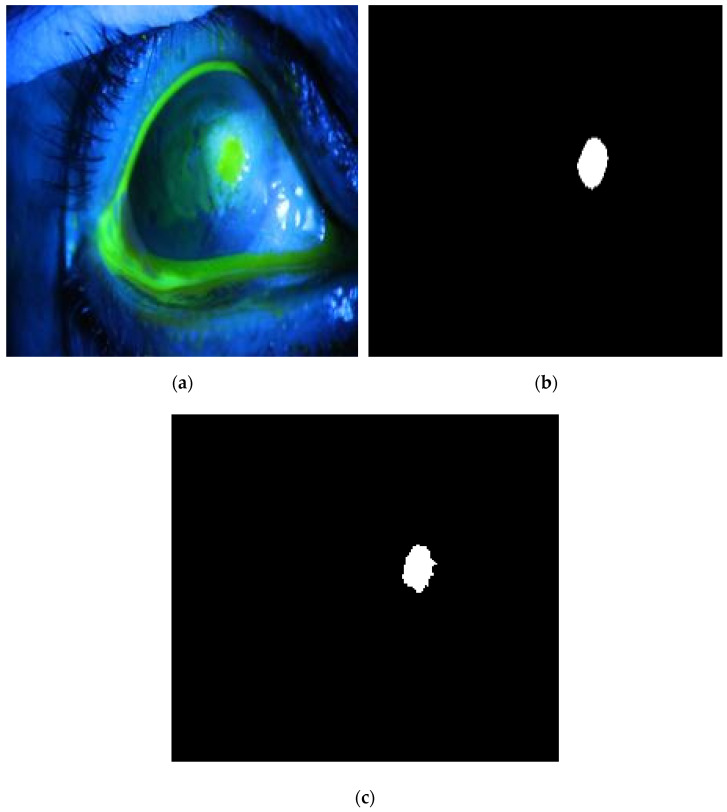
Example 2: Semantic segmentation approach, (**a**) the original image, (**b**) the segmentation output, (**c**) and the ground truth of the corresponding input image.

**Figure 21:**
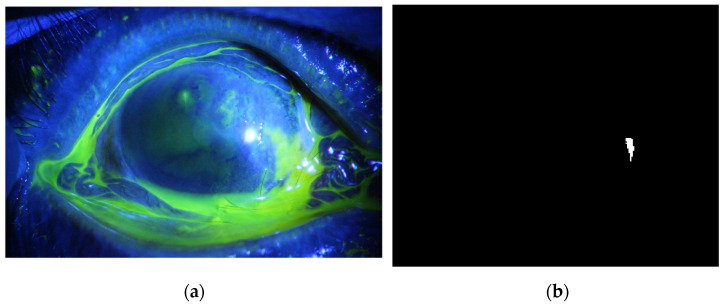

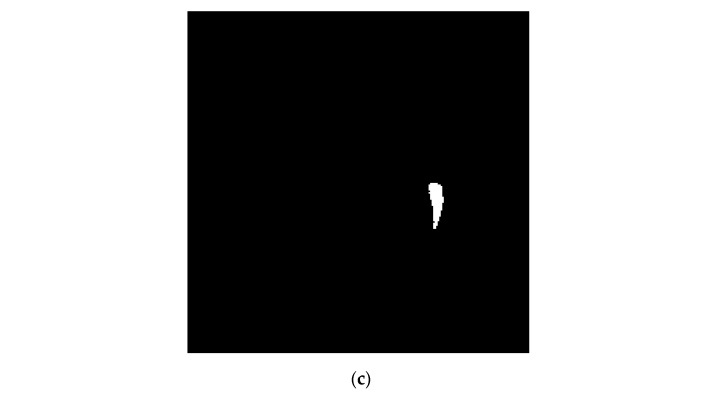
Example 3: Semantic segmentation approach, (**a**) the original image, (**b**) the segmentation output, (**c**) and the ground truth of the corresponding input image.

**Figure 22:**
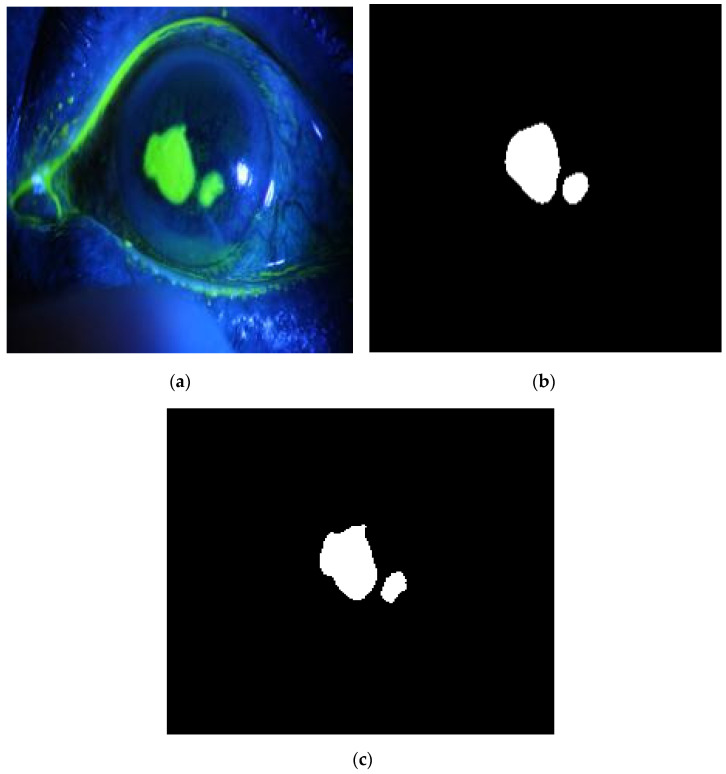
Example 4: Semantic segmentation approach, (**a**) the original image, (**b**) the segmentation output, (**c**) and the ground truth segment.

**Figure 23:**
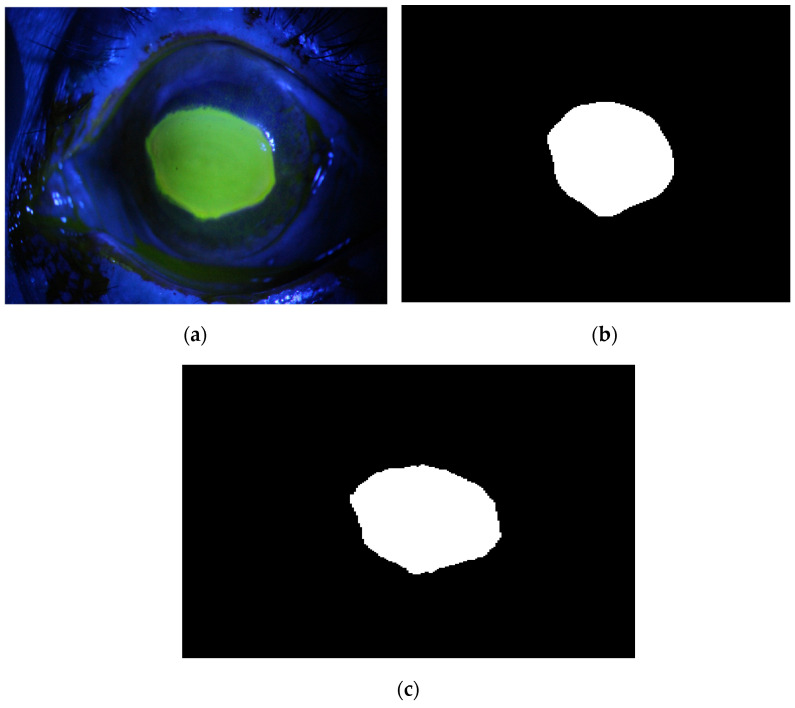
Example 5: Semantic segmentation approach, (**a**) the original image, (**b**) the segmentation output, (**c**) and the ground truth segment.

The proposed method is applied to the dataset. The following Figure 19, Figure 20, Figure 21, Figure 22 and Figure 23 illustrate the output of the deep learning model. Each figure shows the original image and its corresponding ulcer region that is localized by the deep learning model.

Figure 19, Figure 20, Figure 21, Figure 22 and Figure 23 illustrate how sensitive the model is to the ulcer region. In addition, the time required for each test image is less than 1 s, implying that the second proposed method is accurate, sensitive, and fast after building the AI model.

The comparison is performed between the two methods in terms of sensitivity, accuracy, specificity, Jaccard index, and Dice similarity. The Jaccard index expresses the division of true classified pixels over the sum of the number of ground truth pixel and the predicted pixels. It is also defined as intersection of union (IOU), as is clear in the corresponding equation [31]:

$$IOU=\frac{TP}{TP+FP+FN}$$

On the other hand, the Dice similarity defines as two times the area of intersection divided by the sum of the number of pixels predicted and the number of ground truth pixels, and it can be defined as F1 score. The corresponding equation reveals the relation [31]:

$$DSC=\frac{2TP}{2TP+FP+FN}$$

All evaluated matrices are carried out on the same test data, which is formed by 30% of the whole dataset. The number of test data is 107 images. Table 2 depicts the performance of each method on the same images.

Table 2 abstracted the results for both methods and its conclusion of the benefit of deep learning techniques on the traditional image processing tools. In terms of accuracy, specificity, and Jaccard similarity, the second approach is higher than the first one. However, it is less sensitive than the first method. Additionally, the IOU is lower than the image processing proposed method. That comes from the truth; the deep learning approach needs a large dataset to obtain a robust and highly sensitive one by optimizing its training parameters. On the other hand, the time required for the second approach is less than the first approach where the first method requires almost 30s to detect the ulcer region whereas the second strategy is just 1 s for a single test image. Therefore, the second method can be the promised approach for ulcer segmentation in the medical field. Furthermore, building a sensible and reliable model requires training the semantic model on a large dataset.

**Figure 24:**
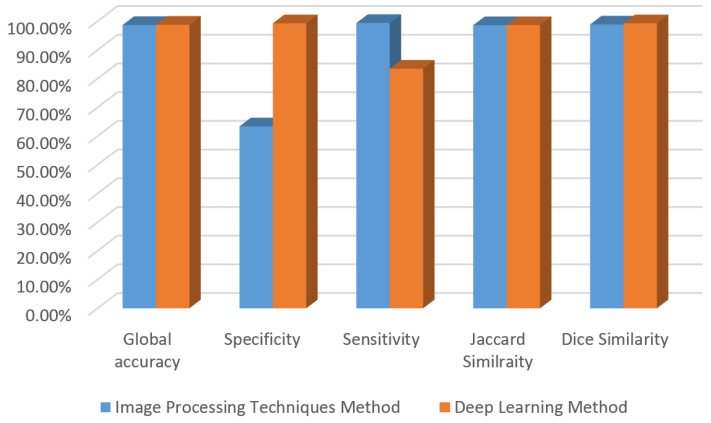
The comparison between the two proposed methods.

Figure 24 describes the performance of each method. Both methods are effective as shown in the corresponding figures. Their IOU and Dice similarity are almost the same. Based on the experiment which is carried out in this paper, the time required to segment ulcers in a single image using AI is just 1 s, where using image processing needs 30 s.

This study compared with literature that used the same dataset. Table 3 describes the performance of both methods in terms of accuracy, sensitivity, specificity, and Dice index.

As illustrated in Table 3, both methods are effective and influence ulcer detection.

## 5. Conclusions

A corneal ulcer is commonly a corneal disease. It causes ocular morbidity due to injury or infection by bacteria, viral, or parasites. Ulcer early diagnosis decreases vision impairment chance. Employing slit-lamp imaging techniques in clinics can be tedious, expensive, and time-consuming. Localization of ulcer regions in slit-lamp images influences the level of diagnoses.

Manual detection needs highly expert physicians, and it is not accurate. Automated segmentation of the corneal ulcer region develops the assessment method and helps diagnose accurately.

This paper proposed two methods to extract the ulcer region automatically. The first approach utilizes image processing techniques with Hough transform to localize the corneal ulcer-affected segment. The second approach is designed based on deep learning algorithms. The two methods are trained and evaluated in terms of performance matrices: accuracy, sensitivity, specificity, Jaccard similarity, Dice similarity, and IOU. The results show the effectiveness of both methods in accuracy, but deep learning is more accurate than image processing. However, image processing is more sensitive to ulcer regions, whereas the deep learning method has higher specificity. This study recommends exploiting the properties of image processing algorithms and artificial intelligence (AI) to guide the residents in extracting the affected ulcer region.

The sensitivity of the AI model can be enhanced using a large dataset to achieve a more sensitive, reliable, and robust model. The two approaches leverage finding appropriate treatment based on the assessment report, which decreases the probability of reaching the visually impaired.

## Figures and Tables

**Table 1:** Performance of semantic deep learning segmentation.

	Global Accuracy	Specificity	Sensitivity
Training Phase	99.75%	99.84%	96.77%
Test Phase	98.8%	99.3%	83.5%

**Table 2:** Comparison between two proposed methods over the test dataset (30% of whole data).

Method	Global Accuracy	Specificity	Sensitivity	Jaccard Similarity	Dice Similarity
Image Processing Techniques Method	98.7%	63.4%	99.4%	98.64%	98.9%
Deep Learning Method	98.8%	99.3%	83.5%	98.655%	99.3%

**Table 3:** Comparison of the proposed method with previous studies.

Study	Accuracy	Sensitivity	Specificity	Dice Index
[10]	88.96%	90.67%	87.57%	88.01%
[12]	-	89.65%	99.7%	89.14%
[13]	-	91.9%		90.93%
**This Study (1st method)**	97.97%	99.8%	63.4%	
**This study (2nd method)**	**98.9%**	**83.5**	**99.3%**	

## Data Availability

The dataset that has been analyzed in this study was derived from the following public domain resource SUSTech-SYSU dataset. Available online: https://github.com/CRazorback/The-SUSTech-SYSU-dataset-for-automatically-segmenting-and-classifying-corneal-ulcers (accessed on 1 May 2022).
